# Terrestrial Laser Scanning for Vegetation Sampling

**DOI:** 10.3390/s141120304

**Published:** 2014-10-28

**Authors:** Jeffrey J Richardson, L. Monika Moskal, Jonathan D. Bakker

**Affiliations:** School of Environmental and Forest Sciences, University of Washington, Box 352100, Seattle, WA 98195-2100, USA; E-Mails: lmmoskal@uw.edu (L.M.M.); jbakker@uw.edu (J.D.B.)

**Keywords:** density, patchiness, forest, remote sensing, grazing, fire

## Abstract

We developed new vegetation indices utilizing terrestrial laser scanning (TLS) to quantify the three-dimensional spatial configuration of plant communities. These indices leverage the novelty of TLS data and rely on the spatially biased arrangement of a TLS point cloud. We calculated these indices from TLS data acquired within an existing long term manipulation of forest structure in Central Oregon, USA, and used these data to test for differences in vegetation structure. Results provided quantitative evidence of a significant difference in vegetation density due to thinning and burning, and a marginally significant difference in vegetation patchiness due to grazing. A comparison to traditional field sampling highlighted the novelty of the TLS based method. By creating a linkage between traditional field sampling and landscape ecology, these indices enable field investigations of fine-scale spatial patterns. Applications include experimental assessment, long-term monitoring, and habitat characterization.

## 1. Introduction

Vegetation field sampling in community ecology has changed little in more than a century; the core metrics of species abundance and richness still provide the foundation of most studies. This is of no surprise, as these measures of community structure are easy to measure by a human observer, requiring only a few simple tools and knowledge of the local flora [1]. Although highly informative, these measures do not assess spatial configuration. The complexity and difficulty of assessing three-dimensional spatial configuration in the field has slowed the integration of landscape ecology, and its emphasis on linking spatial patterns with ecological processes [2], with traditional community ecology. Most methods of assessing spatial configuration are limited to coarse scale information from categorical maps or discrete points [3] that do not align with the scale of ecological field sampling [4]. Furthermore, while field methods such as stem mapping can provide spatial information, they are time consuming, impractical for small plants, and provide only two-dimensional information [5]. Enhanced methods of measuring spatial configuration in the field would thus be a welcome supplement to measurements of vegetation richness and abundance, and could improve our understanding of ecological processes.

Terrestrial Laser Scanning (TLS, also known as Terrestrial LiDAR) can measure the three-dimensional spatial configuration of vegetation at extremely fine scales. The TLS instrument is a portable scanning laser rangefinder that can record millions of discrete three-dimensional points corresponding to the locations of solid objects around the scanner. TLS differs from Airborne Laser Scanning (ALS, also known as Aerial LiDAR) in that it collects a much higher density of points in a much smaller area. The majority of the energy of ALS is directed at the top of the canopy, while the energy of energy from TLS is directed at the bottom or side of the canopy, depending on canopy height and configuration. To date, most TLS studies have sought to use these point data to recreate two-dimensional stem maps [6,7], estimate leaf area index (LAI) and other conventional metrics [8,9], or perform forest inventory [10,11]. Studies are increasingly focused on producing highly detailed three-dimensional models of individual trees [12,13]. These studies focus on using TLS as a tool to arrive at traditional measures of vegetation structure, usually attempting to increase the efficiency and/or precision of these traditional measures.

The novelty of a TLS dataset over any remotely sensed dataset previously available allows for the development of novel ways of quantifying vegetation spatial configuration [14], but little research has demonstrated truly new ways of doing so. A few recent studies have begun to explore novel measures of vegetation density with TLS by examining whether three dimensional sections of space contain vegetation, either through voxelization of TLS point clouds [15] or by calculating a measure of vegetation density in 6.25 m^3^ sections of space [16]. These studies develop methods to correct for the spatial biases inherent to a single TLS scan: occlusion by objects near to the scanner, which block the line of sight to objects further away, and beam divergence, which reduces the effective point density the farther one moves from the scanner. If one is attempting to accurately map vegetation density across a defined landscape, spatial biases present a serious problem, and either correction measures or the addition of additional stitched point clouds from additional nearby scan locations are required [16].

In this study, we present a different approach to quantifying vegetation spatial configuration by accepting the inherent spatial bias of TLS data. Instead of attempting to describe spatial configuration across a landscape, we develop two novel indices of vegetation density and patchiness taken from the perspective of a single point in space, the scan location. These indices leverage the novelty of TLS to create a novel way of quantifying vegetation spatial configuration, but due to their novelty, there is no direct comparisons to metrics obtained using existing field methods. Therefore, rather than performing a traditional validation, we demonstrate the applicability of these indices by comparing them to two traditional measures of vegetation structure within an existing long-term experiment. Finally, we suggest some potential applications of these TLS based indices. These indices give ecologists new and powerful tools to quantify and analyze vegetation spatial configuration at fine scales in the field while allowing spatial linkages to traditional methods of vegetation sampling.

## 2. Methods

### 2.1. Derivation of Indices

**Figure 1.**
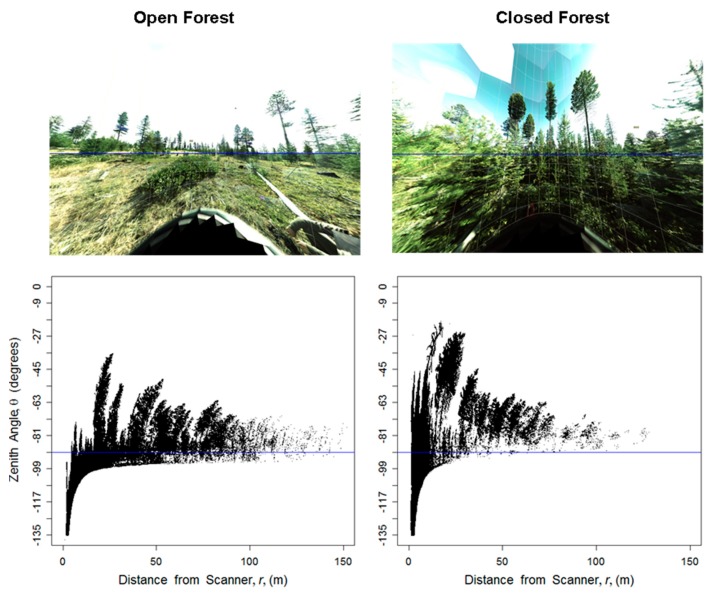
Visualizations of two representative plots, an open forest plot (left), and a closed forest plot (right). The top rows show a photo-mosaic, captured by the TLS scanner, at each plot location. The bottom row shows all points collected by the TLS instrument plotted with their distance from the scanner, *r*, on the horizontal axis and zenith, θ, on the vertical axis. Note that the blue lines correspond to −90° of zenith angle (parallel to ground).

In their raw form, data collected by a TLS instrument consist of millions of discrete points in Cartesian coordinates (X, Y, Z), putting the scanner at the origin (0, 0, 0). Conversion of the X, Y, Z, points to a spherical coordinate system (easily done using a script in R software, for example) allows easier manipulation of the data by describing each point relative to a fixed location. Spherical coordinates describe each point in three-dimensional space by its distance (*r*) from the origin, the zenith angle (θ) measured from directly overhead and relative to a vertical line through the origin, and the azimuthal angle (ϕ) orthogonal to θ. Figure 1 provides sample visualizations of these data in representative open and closed canopy forests. The top row shows a photo-mosaic view of the two forests from the perspective of the scanner. The spherical coordinates *r* and θ are plotted in the bottom graphs. The plots suggest that more points are clumped at small *r* in the closed forest compared to the open forest, and suggest that the distribution of *r* is highly related to vegetation density. However, these plots are not quantitative measures of forest spatial configuration. We suggest that vegetation density be quantified via the Three-dimensional Vegetation Density Index (3DI):

$$3\text{DI}={\tilde{r}}_{\theta_{1},\theta_{2}}$$

where *r̃* is the median distance from the scanner for all points with zenith angle between θ_1_ and θ_2_. Within the specific range of θ, 3DI assesses a three-dimensional space visualized as a cone rotated around its apex. This index can be easily compared among scans.

Variation in vegetation density around the scanner provides an assessment of vegetation patchiness, and thus we also propose the three-dimensional Patchiness Index 3PI:

$$3\text{PI}={\left(\frac{\sigma }{\mu }\right)}_{\theta_{1},\theta_{2}}$$

where σ and μ are the standard deviation and mean, respectively, of the set: *r̃*_ϕ,ϕ+_*_a_* for ϕ [0, 0 + *a*, …, 360 − *a*] where *r̃_ϕ,ϕ_*_+_*_a_* is the median of all *r* with azimuthal angle between ϕ and ϕ+a and zenith angle between θ_1_ and θ_2_, and *a* is the azimuthal angle increment determining the number of bins to use in the index. Note that σ/μ is synonymous with the coefficient of variation.

### 2.2. Application at Starkey Experimental Forest and Range

**Figure 2.**
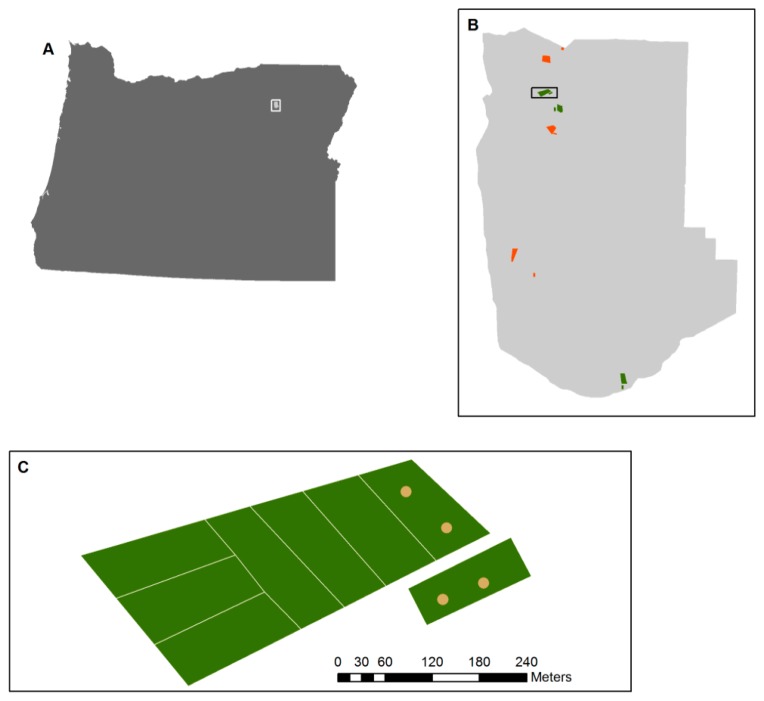
The study area where TLS scans were obtained. (A) shows the state of Oregon with the extent of (B) enclosed by the white box. (B) shows the Starkey Experimental Forest and Range. Red polygons are thinned and burned blocks, and green polygons are untreated blocks. The black rectangle is the extent of (C), which shows the experimental design for an untreated block. Scans were collected at locations represented by yellow dots. The dots within one of the six contiguous rectangles are in the ungrazed plot, while the grazed plot is the unattached rectangle.

TLS data were collected at Starkey Experimental Forest and Range (SEFR), in the Blue Mountains of north-central Oregon in August of 2009 (Figure 2). The TLS scan locations were located within forested areas of a long-term experiment begun in 2000. Three blocks were thinned and burned in 2000, while three others were untreated. In each block, seven experimental treatments were installed: six grazing treatments within a large fenced area, and one unfenced adjacent treatment.

We chose two treatments within each of the six blocks that we believed would show the most variability in vegetation structure: the treatment that had excluded grazing and browsing since 2001, hereafter referred to as ungrazed, and the nearby unfenced treatment that had experienced continuous grazing and browsing by cattle, deer, and elk for the duration of the experiment, hereafter referred to as grazed. In each roughly 0.8 ha treatment, two TLS scan locations were randomly selected under the stipulation that they be >50 m from each other and >20 m from the treatment boundary (Figure 2).

In total, 24 scans were obtained (four scans in each block, two in each grazed and two in each ungrazed treatment). TLS scans were obtained using a Leica Scan Station 2 with the following specifications: a complete scan in 360° of azimuth (ϕ) and 135° of zenith (θ; the 45° of zenith corresponding to the area below the scanner is blocked from the scanner's view) at a point spacing of 2.5 cm × 2.5 cm at 30 m from the scanner. The scanner was controlled using Cyclone software (Leica Geosystems, Heerbrugg, Switzerland) which also allowed the raw data to be exported into a text file for further processing.

We also obtained field data collected by the U.S. Forest Service at the same plots that describe the structural components of the shrub understory. Shrubs were defined by a list of 28 species. These data were collected at multiple subsample locations within each treatment (number of subsamples ranged from 11 to 20). At each subsample location, shrub stem density was counted within a 4 m by 4 m square. Shrub cover was counted along a 10 m line, where a minimum of 2.5 cm of shrub canopy intercepted the transect. Mean values of shrub density and shrub cover for each of the 12 treatments were calculated from the subsamples within that treatment. All statistical analyses were performed in R version 2.11.1 using the “aov” function.

## 3. Results

To apply the two indices described above, we first needed to select an appropriate range of θ for which to summarize *r*. While any number of increments could have been used, we found that dividing the 135° of zenith into 15 bins provided a good overview of the variability; smaller increments masked the overall trends in spatial pattern (results not shown).

**Figure 3.**
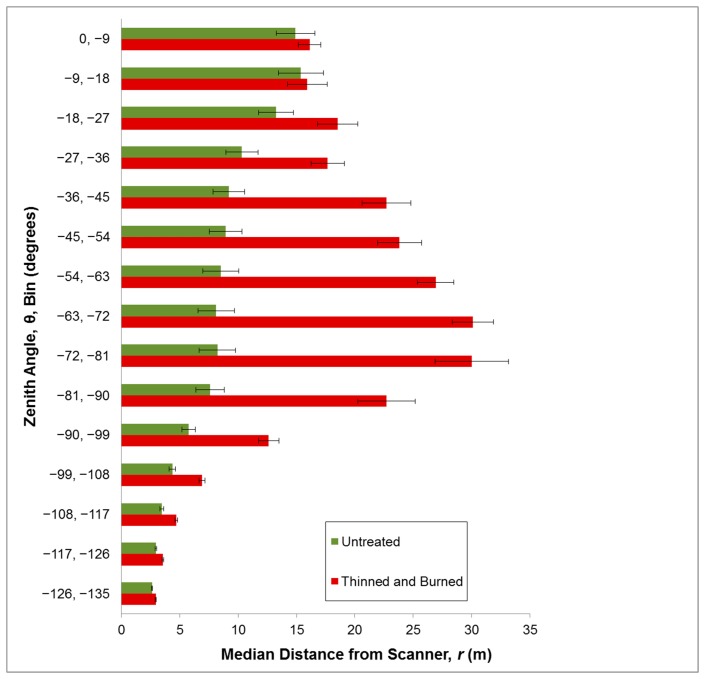
The Three-dimensional Vegetation Density Index (3DI) computed using 15 zenith angle bins for representative plots that were untreated and plots that were thinned and burned. Standard error bars are shown.

**Figure 4.**
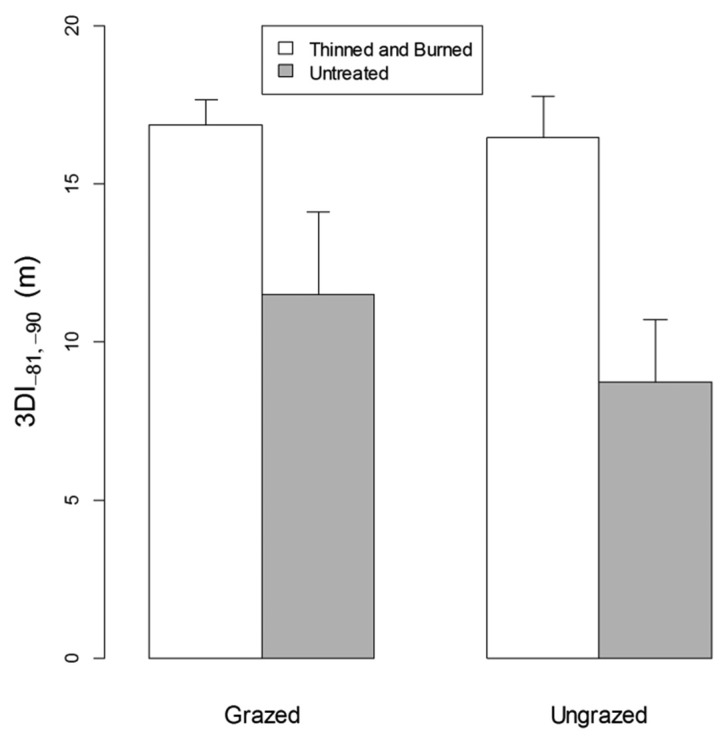
Mean values of the Three-dimensional Vegetation Density Index (3DI) for zenith angles between −81° and −90° for grazed and ungrazed plots that were either thinned and burned or untreated. Standard error bars are shown.

For all angle bins, vegetation density as measured by 3DI was greater in thinned and burned than untreated plots (Figure 3). At the smallest zenith angle (closest to ground) the difference is small because of the relatively short distance the laser travels before it reflects off the ground. We concentrated on 3DI within a θ angle range between −81° and −90° as this range allowed a large swath of vegetation to be sampled while including a minimum of ground points, as the perspective is close to horizontal in all directions. A two-factor split-plot ANOVA (Table 1; Figure 4) indicated that 3DI differed significantly between forest treatments (*p* < 0.01) but not between grazing treatments (*p* = 0.54). The forest treatment × grazing interaction was also not significant (*p* = 0.64).

**Figure 5.**
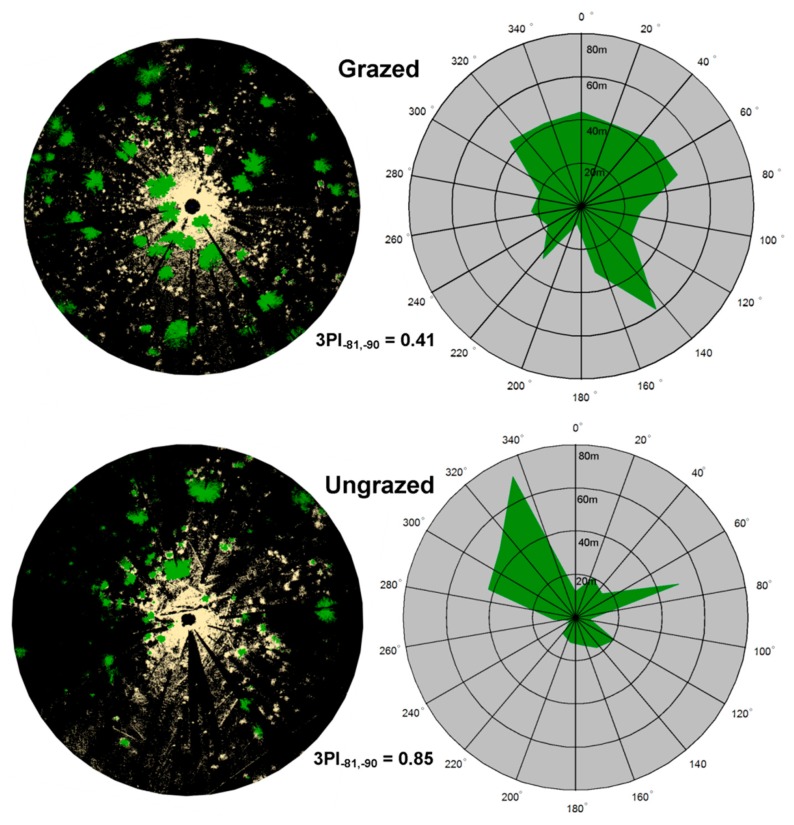
Visual representations of TLS data in a thinned and burned and grazed plot (above) and a thinned and burned and ungrazed plot (below). On the left are overhead views of raw TLS points rendered in Cartesian coordinates. The scan location is at the center, points greater than scanner height (2 m) are shown in green, and points less than scanner height are shown in beige, black areas had no points collected. The radius of each extent is 80 m. On the right are polar plots showing the median *r* for 18 different azimuthal angles ranges. The three-dimensional Vegetation Patchiness Index (3PI) is calculated for each plot using zenith angles from −81° to −90°. Mean and standard deviation used to calculate 3PI are: grazed (4.1, 1.7), ungrazed (23.4, 19.9).

**Figure 6.**
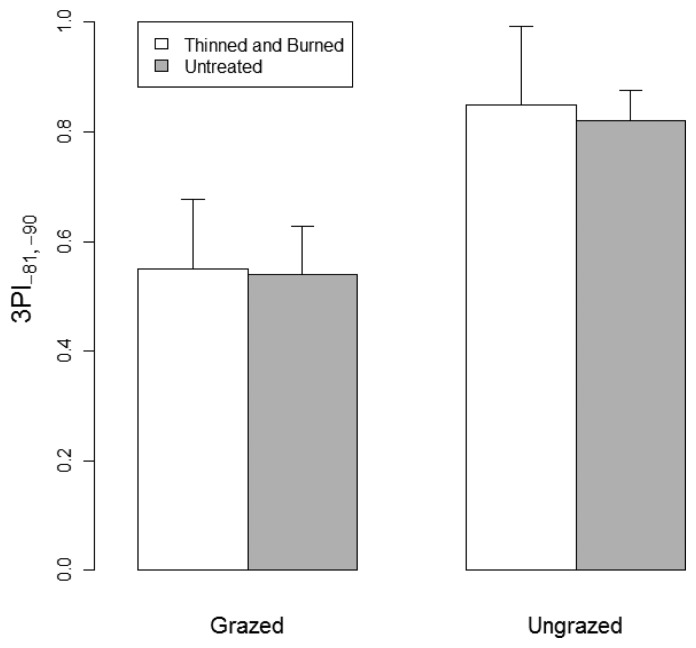
Mean values of the Three-dimensional Vegetation Patchiness Index (3PI) for zenith angles between −81° and −90° for grazed and ungrazed plots that were either thinned and burned or untreated. Standard error bars are shown.

We used the same θ increment to assess vegetation patchiness around the scanner. Figure 5 displays a visualization of patchiness for representative grazed and ungrazed plots that were thinned and burned. Median values of *r* are plotted for each of the 15 bins of ϕ, and 3PI values are given. A two-factor split-plot ANOVA (Table 2; Figure 6) indicated that 3PI did not differ between forest treatments (*p* = 0.86) but tended to differ (*p* = 0.07) between grazed and ungrazed treatments. The forest treatment × grazing interaction was not significant (*p* = 0.94).

**Figure 7.**
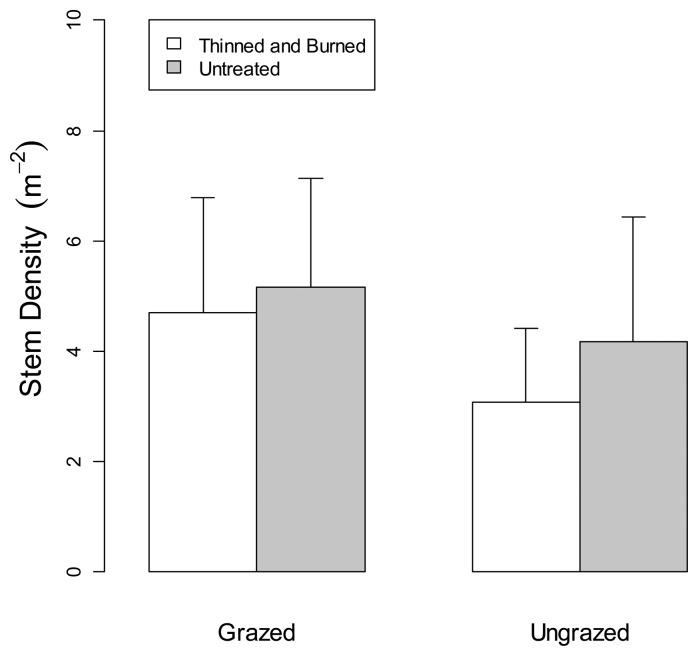
Mean values of field measured shrub stem density in grazed and ungrazed plots that were either thinned and burned or untreated. Standard error bars are shown.

**Figure 8.**
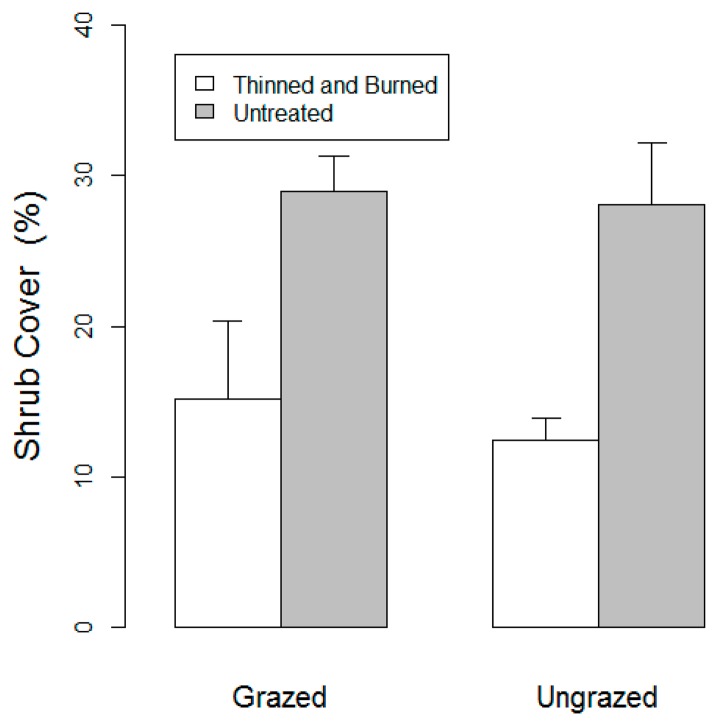
Mean values of field measured shrub cover in grazed and ungrazed plots that were either thinned and burned or untreated. Standard error bars are shown.

Figure 7 shows mean values of shrub stem density calculated from the U.S. Forest Service field collected data. Two-factor split-plot ANOVAs (Tables not shown) indicated that stem density did not differ between forest treatments (*p* = 0.78), grazed and ungrazed treatments (*p* = 0.17), or the forest treatment × grazing interaction (*p* = 0.71). Figure 8 shows mean values of shrub cover calculated from the U.S. Forest Service field collected data. Shrub cover differed significantly between forest treatments (*p* = 0.01) but not between grazing treatments (*p* = 0.68) or the forest treatment × grazing interaction (*p* = 0.83).

## 4. Discussion

The ability to quantify fine scale patterns using a simple, repeatable methodology is of practical use in the myriad experimental designs that investigate vegetation structure. The application of these vegetation density and patchiness indices at SEFR shows how these techniques can be used to investigate quantifiable differences in spatial configuration. Although we did not test pre-defined hypotheses about spatial configuration at SEFR, our results indicate that thinning and burning affected vegetation density but not patchiness, whereas grazing affected vegetation patchiness but not density. However, more detailed understanding of the development of these spatial patterns would require additional study.

The observations based on the field collected measures of vegetation structure (Figures 7 and 8) help to support the validity of the indices developed in this study, while at the same time highlighting the limitations of traditional measures. The measure of shrub cover is most similar to 3DI, as both are responsive to the amount of vegetation present. In both cases, significant differences were observed between treatments that were thinned and burned compared to those that were not. There is an important difference between the two measures: namely that 3DI samples all vegetation types within a given angle bin, while measures of shrub cover don't account for cover of trees or herbaceous vegetation that may also be within a height stratum of interest. The lack of a comparable field based measure to 3PI further highlights the novelty of this new tool for investigating vegetation structure. The intent of these indices is not to replicate these traditional measurements, but rather to summarize TLS data in a new way with the potential to provide new insights relative to those available from traditional measurements.

The unique perspective of these two indices may require a shift in the way remote sensing scientists typically interpret remotely sensed data products. It is common to process remotely sensed data to describe variables in contiguous raster cells across a landscape, such as in LAI derived from ALS [17] or the normalized difference vegetation index (NDVI) derived from Landsat [18]. These products have little to no spatial bias, as each grid cell is exposed to the same energy, either from a laser or the sun. The TLS indices derived here are more akin to field based remote sensing techniques such as hemispherical photographs, which use information from a single point location to estimate variables such as LAI and solar radiation for a single point in space [19], and are therefore spatially biased to that single point in space. Just as in a hemispherical photograph, vegetation that is near to the scanner will strongly affect these indices. Thus, it is key that these indices are treated as a sample of nearby vegetation rather than reflecting a census of the vegetation surrounding the scan location.

## 5. Applications

### 5.1. Long Term Monitoring

The long term nature of the SEFR experiment highlights the power of TLS methods and these indices for long term monitoring. For example, a logical extension would be to obtain repeated scans in the same locations and quantify how density and patchiness change over time. As a benefit, TLS scanning does not modify the environment, as the instrument can be set up outside the area of interest. A TLS based monitoring effort might be able to supplant destructive sampling altogether, as TLS has been used to estimate biomass [20,21] and chlorophyll content [22]. Using a single instrument to quantify spatial configuration and biophysical attributes of vegetation without modifying the community would be a powerful new tool, especially considering the cost and time required for long-term monitoring efforts like LTER and NEON [23,24].

### 5.2. Integration with Traditional Field Sampling

An investigator seeking to integrate TLS-based methods with traditional sampling might use transects or Daubenmire style quadrats to assess species richness and abundance at each TLS scan location. This is especially efficient because there is a period of time after the scan commences during which data could be collected in areas already scanned. An index such as Shannon's Diversity would provide a single metric which could then be easily compared or integrated with the TLS derived indices.

**Figure 9.**
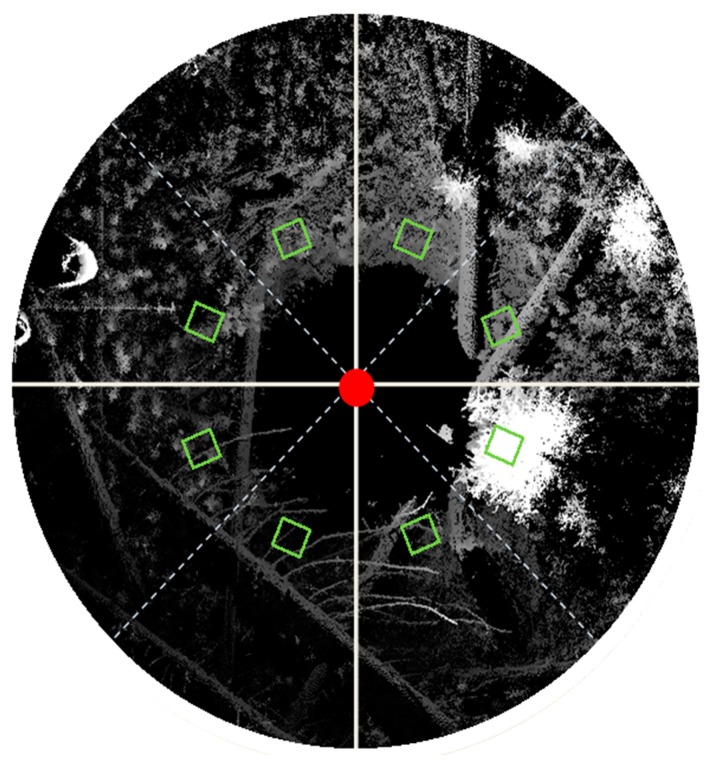
Suggested sampling strategy for linking field measurements of species abundance and richness with vegetation spatial configuration indices (3DI and 3PI). The image is an overhead view of a TLS point cloud, rendered by height with the tallest points white and the lowest points black. Superimposed on the image are the TLS instrument location (red dot), quadrants of the scanning area (delineated by solid lines), vegetation transects (dotted lines), and Daubenmire style vegetation quadrats (green squares).

The area surrounding the scanner could also be separated into quadrants in order to link even finer scale patterns in richness and abundance to vegetation density and patchiness. An example sampling strategy is shown in Figure 9. 3DI and 3PI can be easily modified to assess density and patchiness within a quadrant by calculating each index using the totality of the data within the quadrant, and in the case of 3PI, dividing each quadrant into a discrete number of ϕ bins. Investigators should be wary of pseudoreplication when dividing a plot into multiple sub-samples for experimental assessment [25], but the technique would be robust for studies where examining fine-scale spatial variability of plant community structure is the principal focus.

It is also possible using TLS to collect point clouds corresponding to the exact spatial location of quadrats or belt transects rather than the neighborhood represented by a quadrant. The indices developed in this study should not be used to assess these point clouds, as the indices are built on the unique geometry of point clouds collected using the full field of view of the TLS scanner. The main assumption of these indices is that each increment of ϕ contains an equal number of laser pulses with equivalent spacing and geometry. Laser pulse density declines with distance from the laser; to be comparable, a field collected belt transect would need to increase in width as distance from the scanner increased. Other methodologies, such as biomass estimation based on estimated canopy volume [26], may be more appropriate for direct comparison of TLS point clouds clipped to the extent of field measurements.

### 5.3. Applications for Habitat Characterization

Another clear application of these methods is habitat characterization, one of the most common ecological applications of structural measurements of vegetation. Unlike airborne LiDAR, TLS has yet to be applied to this purpose [14]. The fixed location of the TLS produces a three-dimensional representation of the surrounding space, similar to how an organism may perceive the space. For example, the experiment at SEFR sought to measure the influence of ungulate grazing on forest structure. The indices provide a quantification of the patterns an ungulate may experience in those spaces, which may then inform the resulting ecological processes that affect the observed results. If the significantly greater patchiness observed in the grazed treatment is attributed to modification by ungulates, the perspective of the TLS, 2 m above the ground and at ungulate eye level, may aid in understanding how ungulates affect vegetation structure when the complete long-term results of this experiment are later analyzed.

By adjusting the ϕ and θ bins, investigators can tailor the quantification of vegetation structure to an appropriate scale for their organism(s) of interest. While ungulate grazers may interact with their environment over relatively coarse scales that leverage the entire field of view of the TLS, smaller organisms such as insects may modify their environments at very fine scales, thus requiring many small ϕ bins, while canopy dwelling organisms may necessitate that only θ bins above the horizontal plane are included in analysis. Furthermore, if a scan with a high point density using the full field of view is performed, much of these decisions and analyses can be performed after the field campaign by selecting subsets of the full TLS dataset.

### 5.4. Applications to Non-Forested Systems

While we used a forest ecosystem to demonstrate the vegetation density and patchiness indices, they would be robust in many different ecosystems. Grasslands, for example, are difficult to assess structurally because the plants are so small and numerous. Small scale measurements of density are often restricted to visual estimates of percent cover [27], while patchiness estimates require remotely sensed data that may be too coarse in scale [28]. TLS can provide measurements of density and patchiness because its laser beams are focused enough to sample fine structures such as individual blades of grass. Practitioners may want to modify the increments and ranges of θ and ϕ as well as constraining the range of *r* to better suit their system and accurately sample variation at the scale of interest. Grasslands, for example, may more efficiently be sampled at locations very close to the scanner but with a larger number of ϕ increments because vegetation varies at a finer scale.

### 5.5. Avenues for Future Research

One significant research need is how these indices, and TLS based methods in general, can be applied in areas with highly sloped terrain. On a slope, the TLS instrument cannot sample equally in all directions of ϕ. On the uphill side, more laser beams will reflect off the ground, while the opposite is true on the downhill side. Without correction, this can affect both indices, as point density will be higher on the uphill side regardless of the vegetation present. In this study, the plot locations were all located on relatively flat topography, but the effects of slope can still be seen. The open forest in Figure 1 provides an example of non-flat terrain; the photomosaic shows ground sloping slightly uphill to the left and downhill to the right. The pattern is also reflected in the plots of *r* and θ, where distinct “ground lines” can be seen. Slopes should be avoided while using these methods, unless modifications and corrections are developed. For example, it is possible to angle the scanner to match the slope as is sometimes performed with hemispherical photography [29] or to construct a digital elevation model from the raw points and adjust the indices in post-processing. Constraining the area sampled to only short distances away from the scanner may limit the effect of slope.

We encourage other researchers to creatively develop TLS-based methodologies. The vegetation density and patchiness indices developed in this study are a first step toward realizing the power of the instrument as a tool for radical innovation in ecological data collection.

## 6. Conclusions

In conclusion, we have shown how an emerging technology, TLS, can be used to produce precise measurements of vegetation spatial configuration through two new indices of vegetation density and patchiness. The fine scale at which the TLS operates allows a linkage between traditional vegetation sampling and landscape ecology, which has been mostly constrained to coarse scale examination of raster and point data. TLS methodologies of vegetation sampling are also well suited for experimental assessment, long-term monitoring, and habitat characterization, and integrate well with traditional methods of sampling vegetation communities. These indices can be applicable in many different ecosystems and represent one of the first practical methods of assessing spatial configuration in three-dimensions at fine scales in the field.

## Figures and Tables

**Table 1.** Split-plot analysis of variance for the Three-dimensional Vegetation Density Index (3DI). Block Treatment is Thinning and Burning/untreated, Plot Treatment is Grazed/ungrazed. Note that the mean of two 3PI values were used in the ANOVA, computed using two separate scans within each replicate.

**Source**	***df***	**Sum of Squares**	**Mean Square**	***F*-value**	***p***
***Whole Plot Factor***					
**Block Treatment**	1	128.69	128.69	44.55	<0.00
**Error**	4	11.55	2.89		
***Subplot Factors***					
**Plot Treatment**	1	7.39	7.39	0.44	0.54
**Block X Plot**	1	4.28	4.28	0.25	0.64
**Error**	4	67.38	16.84		

**Table 2.** Split-plot analysis of variance for the Three-dimensional Vegetation Patchiness Index (3PI). Block Treatment is Thinning and Burning/untreated, Plot Treatment is Grazed/ungrazed. Note that the mean of two 3PI values were used in the ANOVA, computed using two separate scans within each replicate.

**Source**	***df***	**Sum of Squares**	**Mean Square**	***F*-value**	***p***
***Whole Plot Factor***					
**Block Treatment**	1	0.0011	0.0011	0.35	0.86
**Error**	4	0.12	0.031		
***Subplot Factors***					
**Plot Treatment**	1	0.25	0.25	6.25	0.07
**Block X Plot**	1	0.00023	0.0023	0.0056	0.94
**Error**	4	0.16	0.040		
